# Progress in State Medicine

**Published:** 1900-09-29

**Authors:** 


### Progress in State Medicine.

### PREVENTION OF TUBERCULOSIS.

(*Continued from page* 339.)

Niven[5] attributes the great reduction in mortality from phthisis during the last fifty years to improved hygiene, sanitation, cheaper food and clothing, and the greatly increased knowledge of the life history of the tubercle bacillus. Counteracting these would always be, overcrowding, alcoholism, and dirt. The strongest weapon to fight with was an educated rigorous cleanliness. This power of educating lay, firstly, in the hands of the medical profession; secondly, with the sanitary authorities. All medical schools should, as part of the curriculum, insist on students acquiring a knowledge of all the means of counteracting the propagation of tubercle, of staining bacilli, and of diagnosing obscure illnesses by the use of minimal doses of tuberculin. In the poorer districts the onus lay with the sanitary authorities. Much has been done in Manchester by the distribution of handbills, setting forth precautions to be taken, by popular lectures, voluntary notification, and thorough disinfection. Subsequently it was not successive disinfections which were required, but sustained cleanliness. Hospitals should be provided, if possible, by the guardians of the poor for tuberculous patients. For the advanced cases isolation is imperative, but every means must be taken to prevent any idea of pauperisation of the tuberculous poor.

McVail[6] likewise mentions the great decrease in tuberculous affections. Since the decennium 1851-60, the diminution in the tubercular group of diseases has been 39 per cent., and in the particular form of pulmonary consumption over 45 per cent. Tabes mesenterica in children under one year has increased 27 per cent. With this exception the improvement is doubtless due partly to more accurate diagnosis, but principally to improved house construction, better ventilation, systematic refuse removal, and greater domestic and municipal cleanliness. Still much remains to be done, especially in large towns, in checking the smoke nuisance, both as regards factories and private houses. The danger of infection from dried sputum, especially in large manufacturing towns, might be greatly lessened if the pavements of the popular working class parades were washed on Monday morning. Children, owing to the cow being dangerous, more as a milk giver than meat giver, run unnecessary risk of infection. Popular apathy in regard to sterilisation is very great, and objection is also taken to the taste of boiled milk. Whether the granting of legislative powers to great municipalities to restrict the sale of raw milk within their bounds would be interfering with the liberty of the subject, is a questionable point. Failing this, systematic search for tubercle bacilli in the milk supply, and when found, prosecutions instituted under the Public Health Acts for the sale of unwholesome food. Systematic voluntary notification of cases by medical men is at the present time preferable to compulsory notification. Thus unsatisfactory conditions of overcrowding, want of ventilation, uncleanliness, could be dealt with, and disinfection carried out, when needed, by the public health officials. Isolation will be easier when the public more fully understand the nature of the disease. The increased railway facilities, cheap tickets, and the pushing of tram lines to the furthest suburbs of our big towns, all tend to promote a healthier condition in our artisan classes.

Philip[7] points out that the open-air treatment is not such a recent method as is generally supposed. Bodington, in 1840, published an essay, in which the open-air methods were advocated for the cure of pulmonary consumption, and, again, a few years later MacCormac wrote a work, "Consumption as Engendered by Rebreathed Air."

Philip, whilst not ignoring the effects of climate, both favourable and prejudicial, considers that neither mere elevation, temperature, dryness or moisture of the atmosphere are essential in the open-air treatment. The character of the soil is important, the dryer the soil, other things being equal, the better for the patient. There is danger of too little exposure rather than too much. If suitably clothed there is no risk whatever, even in rain, snow, wind, or even mist. Preferably the patient should sleep out of doors, but if this is not possible, the bed should be placed close up to the open window. If attention is given to keeping the peripheral circulation well maintained, no risk is incurred from the night air. In those cases with a swinging temperature rest is indicated, followed by gradually increased walking exercise. The great amount of fresh air and moderate exercise creates a good appetite, which does away with all idea of "forced feeding." Three or four good meals a day at distributed intervals are sufficient, but nothing should be given between times. The body should not be over-clothed. At no season should more than a single shirt and an under woollen garment be permitted, in addition to the upper suit or dress. In addition to, and often in conjunction with, the exercise, some sort of chest expander is beneficial. The skin of the tuberculous patient should be freely cleansed, either with baths or sponging, once or twice daily. Early diagnosed cases reap the most benefit. The benefits derived from a course of treatment are: Increased weight, loss of cough and expectoration, cessation of night sweats, improved appetite, and regular temperature.

Otis,[8] outlining a systematic scheme for the prevention of tuberculosis, advocates compulsory notification of all cases of that disease; failing that, voluntary notification should be encouraged. In addition to instructing the general public in preventitive means, disinfection or renovation of premises occupied by a consumptive, or in which a consumptive has died, should be compulsory, before they can again be let or occupied. Free hospitals, in or near the big towns, should be provided for the poor, with sanatoria in the country for the open-air treatment, and isolation hospitals for the advanced cases. Free examination of sputum for bacilli by the sanitary authorities should be instituted. The city milk supply must be under control, and all cows from which the milk is obtained be certified free from disease by the tuberculin test, such test to be repeated at stated intervals. Knopf[9] is not in favour of compulsory notification, since the danger to the community is not so much from the bedridden patient, but from the consumptive subject who, although expectorating tubercle bacilli freely all day long, is able to go about his work. He advocates the inspection by the health authorities of all institutions which admit cases of phthisis to see that all preventitive precautions are observed. In the event of a patient disregarding advice as to the proper disposal of his sputum, he should be notified to the authorities as dangerous, and isolated. Herman Weber[10] points out that the mere inhalation of tubercle bacilli does not always lead to tuberculosis, as a healthy mucous membrane would expel them in the sputum before they had done any harm, and a strong constitution render them inert. Similarly, not every tubercular or scrofulous gland causes this disease, as may be seen from so many so-called scrofulous children growing up under favourable circumstances into strong, healthy people. He also advocates that rooms occupied by consumptives should be disinfected before they are again inhabited, and also that their clothes should be similarly treated before being otherwise disposed of. The presence of tuberculous servants in the house is a source of infection, the danger being greater to children should they come in contact with them. Mixed food of meat, milk, products of corn and other vegetables, is preferable to an exclusion of vegetable or meat diet. Sufficiency of good food is one of the most powerful means of prevention and cure in tuberculosis. Most stringent regulations should be enforced in the slaughtering of cattle, and all carcases inspected by qualified inspectors. Dairies should be established which would obtain their milk only from cows which have been tested by tuberculin. Pure milk is a necessity for both poor and rich alike.

Some interesting experiments,[11] conducted by Professor Axe for the British Dairy Farmers' Association, point to the fact that cows leading purely an outdoor life are much freer from tuberculosis than those kept constantly confined. In a herd of 41 cows living entirely out of doors, not a single one reacted to the tuberculin test; whilst out of 36 animals kept in constant confinement, 90·9 reacted. On the other hand, a report, issued by the Department of Agriculture of the University of Aberdeen, states that out of 240 cattle tested, 24 of those which gave no reaction were found post mortem to be tuberculous. The same report also confirms the opinion that tuberculin loses its properties when kept for any length of time, but when properly used is a reliable diagnostic in cattle. Cows kept continually in the open air require more food and give less milk than those confined, but this is compensated for by the fact that the milk of the former is much cleaner, and that they do not require so much looking after.

[5] Lancet, Feb. 3, 1900. [6] Scot. Med. Sur. Jour., vol. vi., No. 1. [7] Ibid. [8] Boston Med. and Surg. Jour., Sept. 21. [9] New York Med. Jour., Sept. 23, 1899. [10] Tuberculosis, vol. i., No. 2, Jan., 1900. [11] Brit. Med. Jour., Oct. 28, 1899.

